# Accuracy of Tissue and Sonication Fluid Sampling for the Diagnosis of Fracture-Related Infection: Diagnostic Meta-Analysis

**DOI:** 10.7759/cureus.14925

**Published:** 2021-05-09

**Authors:** Elsiddig A Ahmed, Maya K Almutairi, Amjad T Alkaseb

**Affiliations:** 1 Department of Orthopedics and Traumatology, Prince Mutaibb bin Abdulaziz Hospital, Sakaka, SAU; 2 College of Medicine, Jouf University, Aljouf, SAU

**Keywords:** fracture-related infection, tissue culture, sonication fluid sampling, diagnosis, accuracy

## Abstract

Evidence shows that tissue sample culturing is the gold standard approach for diagnosing fracture-related infection (FRI). Sonication fluid sampling has also been reported to be efficacious and superior to tissue sample culturing with prosthetic joint infection. However, data from the current literature are not enough to validate this hypothesis for FRI. We conducted a meta-analysis to validate the diagnostic accuracy of tissue sample culturing and sonication fluid aspiration and to find which one is superior to the other. An extensive search through the relevant databases was conducted to obtain all the relevant studies. We have included 13 relevant studies, including nine retrospective cohorts and four prospective ones. The overall pooled estimates of sensitivity, specificity, and diagnostic odds ratio (DOR) of tissue sampling culture in diagnosing fracture-related infections were 98% (95% CI, 92% to 99%), 38% (95% CI, 23% to 56%), and 25 (4 to 154), respectively. The overall pooled estimates of sensitivity, specificity, and DOR of sonication fluid sample culture in diagnosing fracture-related infections were 86% (95% CI, 79% to 92%), 98% (95% CI, 93% to 100%), and 353 (78 to 1598), respectively. No significant risk of bias was found regarding the sensitivity and specificity among studies investigating both modalities, however, significant heterogeneity was noticed. Sonication fluid sampling can be used to confirm FRI while tissue sampling can be used to exclude it and both modalities should be combined for obtaining the most accurate outcome.

## Introduction and background

Complications following bone trauma are relatively common and constitute around 14% [1]. Previous estimates show that the occurrence of fracture-related bone infections (FRI) and osteomyelitis are relatively common following 1% closed fractures and around 10%-30% open fractures [2-4]. Such events are attributable to many factors as perioperative contamination, damage to the underlying related vasculature, or wound contamination when the fracture occurs [5-6]. Management might be challenging as the inoculated bacteria can build up an extra-cellular matrix that can intervene against these bacteria and the immune system and many antibiotics [4]. Late diagnosis and improper management can furtherly lead to the development of serious complications as sepsis, delayed, or failed healing of the affected bone, which can lead to amputation, permanent disability, and impaired daily functions [6-7].

Therefore, it is essential to initiate the diagnosis and management of any potential infections. The diagnosis of FRI is based on clinical, microbiological, and serological examinations. However, there are no unified protocols for this process, unlike the pre-existing prosthetic joint infections diagnostic protocols on which the current FRI diagnostic modalities are usually based [8-9]. Many diagnostic modalities have been previously reported for the detection of post-fracture bone infections. The most commonly used modality is culture from an open bone biopsy, which is even marked as the gold standard in this case [10-11]. Other modalities include measuring erythrocyte sedimentation rate, white blood cell counts, C-reactive protein, histopathology, culture from deep wounds, percutaneous culturing of bones, and sonication of the aspirated fluids that can be furtherly investigated for the presence of the infecting microorganisms [5,10,12-13]. However, no sufficient evidence could be found for validating these modalities [10,12], and cultures based on open bone biopsies would remain the gold standard.

Onsea et al. summarized the results of nine studies that investigated the validity of cultured sonicated fluids, PCR, and histopathology examinations [14]. Although the study was comprehensive for all the potentially related studies in this field back in 2018, the results were not conclusive and the authors admitted that further evidence is needed, as none of the diagnostic modalities could be marked superior to the others, according to the evidence obtained from the five included investigations. Consequently, we think that a diagnostic meta-analysis would be useful for the validation of tissue-culture and sonication fluid sampling in the detection of FRI based on previous and recent data. That is why we conducted the current study.

## Review

Search strategy and study selection

Our study used the Preferred Reporting Items for Systematic Reviews and Meta-Analyses (PRISMA) checklist [15]. An electronic database search for suitable studies was performed towards January 15, 2021, in six databases, including Google Scholar, System for Information on Grey Literature in Europe (SIGLE), Scopus, Web of Science (ISI), PubMed, and New York Academy (NYAM) of Medicine databases. We used the following search term: (Sonication OR soniced OR "sonication fluid" OR "sonication culture") AND (fracture OR implant failure) AND ("orthopedic infection") and (Sonication OR soniced OR sonication fluid OR sonication culture) AND (fracture OR implant failure) AND (orthopedic infection). We performed a manual search for the collection of missed papers collected via manual search trials in Google Scholar and references of the included papers [16].

On one hand, we included all relevant original publications reporting the diagnostic accuracy of tissue sampling or the diagnostic sonication fluid sampling that are related to infectious fracture. On the other hand, we excluded studies with any of the following: 1) no history of fractures; 2) diagnostic accuracy measures were not reported; 3) in vitro or non-human subjects; 4) duplicate/overlapped data; 5) abstract-only/conference articles, books, dissertations, reviews, editorials, letters, author responses, and comments.

Title and abstract screening were done by three independent reviewers for identifying eligible papers. This was followed by a full-text screening by three reviewers to ensure the suitability of the included papers. Any disagreement was done by discussion and consulting the senior author, whenever needed.

Data extraction

Data extraction was conducted by three authors, with a fourth one performing data checking for the accuracy of the data. The data extraction sheet consisted of three parts: the first part includes reference ID, recruitment year, age (mean (SD)), male sex, method of osteomyelitis diagnosis, study design, and sample size, the second part includes outcomes of interest (diagnostic accuracy measures), and the third part included studies’ quality assessment. The senior author resolved any disagreements that were raised through discussion.

Quality assessment

The evaluation of the risk bias was carried by three independent reviewers. For this purpose, the revised Quality Assessment of Diagnostic Accuracy Studies (QUADAS-2) tool was used [17]. Any differences that were raised were solved by discussion.

Statistical analyses

Statistical analyses were performed with STATA (version 16 IC; Stata Corporation, College Station, TX). The pooled sensitivity, diagnostic odds ratio (DOR), and specificity with 95% confidence intervals (CI) were calculated. The random-effects model was used to pool the estimated effects. Other diagnostic accuracy measures included a summary receiver operating characteristic (SROC) curve and a summary with a positive likelihood ratio (PLR) as well as a negative likelihood ratio (NLR) [18]. A calculated PLR value higher than 10 and a calculated NLR value lower than 0.1 were observed to provide convincing diagnostic evidence [18-20]. The clinical or patient-relevant utility was reported using a Fagan plot (Bayes Nomogram) to demonstrate the diagnostic test. The likelihood ratios to calculate the post-test probability (PTP) based on Bayes’ theorem were used accordingly [21]. Moreover, a likelihood ratio scattergram [22] and probability modifying plot [23] were also plotted for further investigation of the diagnostic accuracy.

Testing for publication bias was performed using “a regression of diagnostic log odds ratio against 1/sqrt (effective sample size), weighting by effective sample size, with P-value <0.10 for the slope coefficient indicating significant asymmetry,” as described by Deeks et al. [24]. Moreover, evaluating the heterogeneity among the included studies was evaluated by Q statistic and I-squared test by describing the variability in the effect [25-26]. Significant heterogeneity is considered when the P-value was less than 0.1 or the I-squared was more than 50% [27-28]. Publication bias could not be assessed since the included studies are less than 10 [29].

Study characteristic

**Figure 1:**
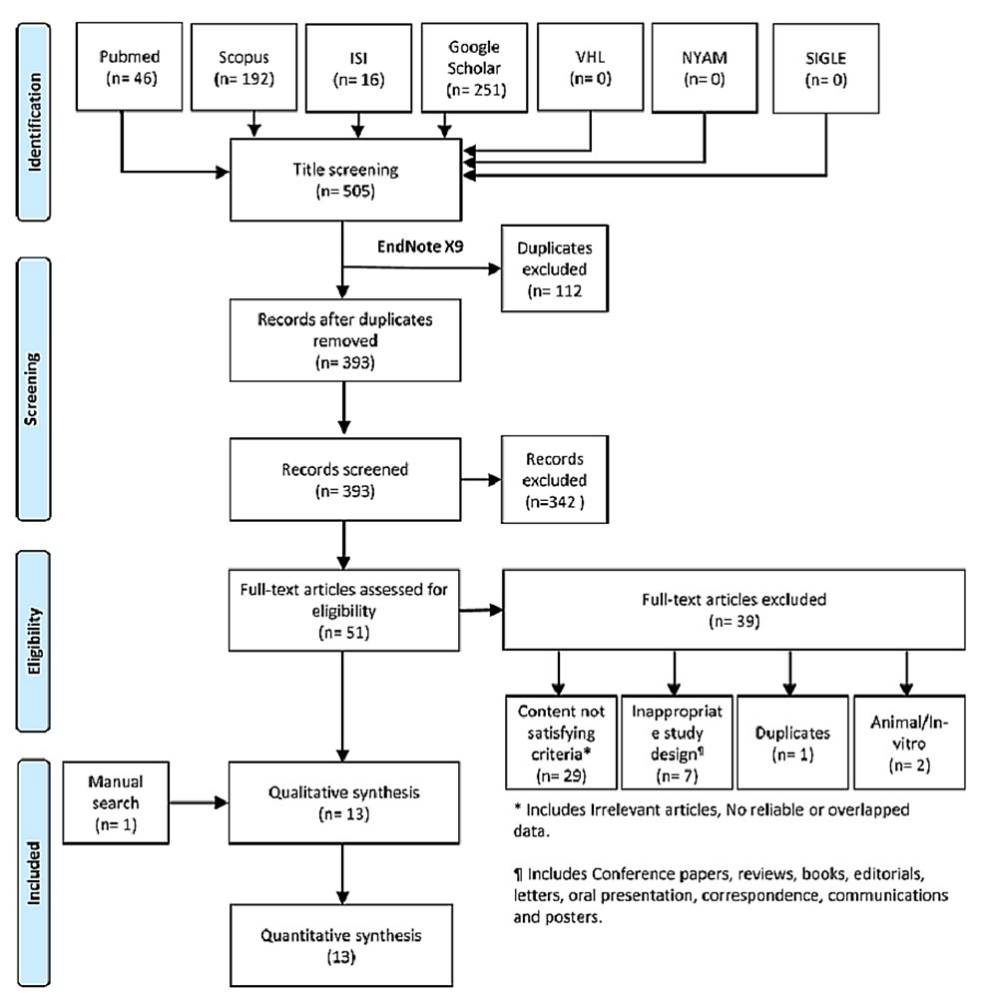
PRISMA flow chart showing the process of the review PRISMA: Preferred Reporting Items for Systematic Reviews and Meta-Analyses

Our search in the seven databases resulted in 505 records and following the removal of 112 duplicate records by EndNote software (Clarivate Analytics, Philadelphia, Pennsylvania); 393 studies were available for full-text screening. Out of these, only 51 full texts were retrieved for assessing their suitability for inclusion in the current study. Finally, 13 papers were finally analyzed, including one paper that was obtained from the manual search of references (Figure 1).

There were nine retrospective cohorts and four prospective ones. The sample size among the included studies was variable and ranged from 27 to 317. The mean female percentage was 56.1% and ranged from 16.7% to 77.8%. The diagnostic accuracy measures for sonicate fluid culture and tissue culture were summarized in Table 1.

**Table 1:** Summary of the included studies CI: confidence interval, NR: not reported, SD: standard deviation,

Study ID	Study design	Sample size	Total of infected patients	Age (years)	Female gender (%)	Sonicate fluid culture				Tissue culture			
				Mean ± SD (range)		Sensitivity		Specificity		Sensitivity		Specificity	
						%	95% CI	%	95% CI	%	95% CI	%	95% CI
Puig-Verdié/2012/Spain [30]	Retrospective cohort	317	109	62.7	58.4%	89.9	NR	99	NR	67	NR	99.5	NR
Yano/2014/Brazil [31]	Prospective cohort	180	125	(12-62)	47.2%	90.4	83.9–94.4	90.9	80.4–96	56.8	47.6–65.3	96.4	87.7–99.6
Portillo/2015/Spain [32]	Prospective cohort	75	39	NR	NR	87	73–96	100	90–100	59	42–74	100	90–100
Esteban/2008/Spain [33]	Prospective cohort	31	17	NR	NR	94.7	NR	50	NR	84.2	NR	100	NR
Renz/2018/Germany [34]	Prospective cohort	51	38	56±49.6	64.7%	84	NR	100	NR	66	NR	100	NR
Banousi/2020/Greece [35]	Retrospective cohort	243	91	64.94±17.4	50.6%	91	83–96	100	97–100	43	33–54	100	97–100
Bellova/2019/Germany [36]	Retrospective cohort	257	145	NR	67.3%	69	NR	90.2	NR	62.8	NR	92.9	NR
Fernández-Sampedro/2017/Spain [37]	Prospective cohort	130	130	66±13	49.2%	84.6	77.2–90.3	99.5	98.1–99.9	62.3	53.4–70.7	99.7	98.5–100
Finelli/2020/Brazil [38]	Prospective cohort	54	47	35.5±11.2	16.7%	97.6	NR	71.4	NR	89.4	NR	71.4	NR
Sebastian/2018/India [39]	Prospective cohort	40	27	(14-80)	45.0%	92.6	75.7–99.1	100	75.3–100	66.7	46–83.5	100	75.3–100
Tani/2017/Greece [40]	Prospective cohort	114	61	(45-88)	74.6%	77.04		98.11	89.9–99.9	55.73	NR	94.34	84.3–98.8
Van Diek/2017/Netherlands [41]	Retrospective cohort	233	75	66.1±2	65.2%	47	35–59	99	96-100	68	56–78	80	74–86
Vergidis/2011/USA [42]	Prospective cohort	27	9	60	77.8%	89	52-100	100	85-100	55	21-86	93	76-99

Risk of bias

**Figure 2:**
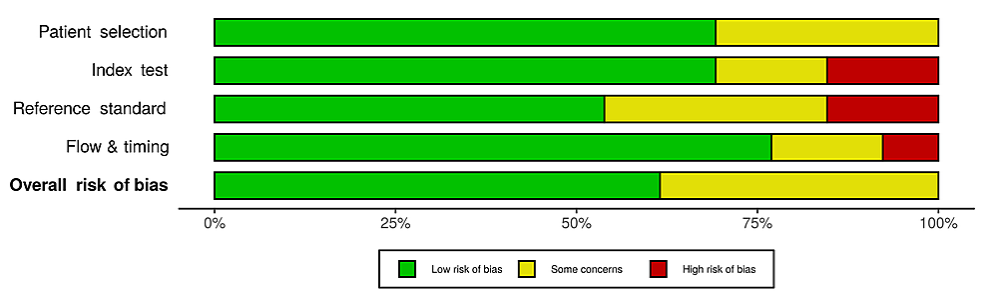
Risk of bias assessment - the revised Quality Assessment of Diagnostic Accuracy Studies (QUADAS-2) tool

**Figure 3:**
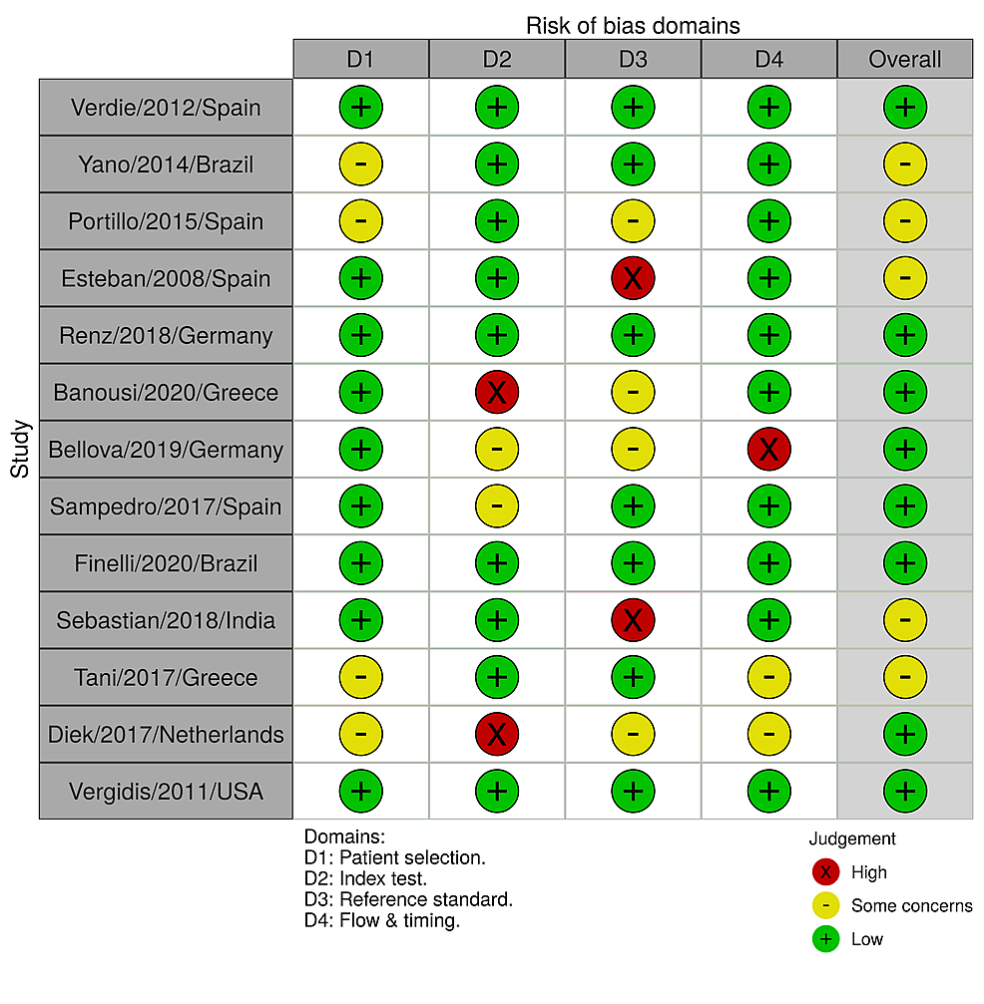
Risk of bias assessment - the revised Quality Assessment of Diagnostic Accuracy Studies (QUADAS-2) tool Puig-Verdié/2012/Spain [30] Yano/2014/Brazil [31] Portillo/2015/Spain [32] Esteban/2008/Spain [33] Renz/2018/Germany [34] Banousi/2020/Greece [35] Bellova/2019/Germany [36] Fernández-Sampedro/2017/Spain [37] Finelli/2020/Brazil [38] Sebastian/2018/India [39] Tani/2017/Greece [40] Van Diek/2017/Netherlands [41] Vergidis/2011/USA [42]

Two-thirds of the included studies had an overall low risk of bias while the remaining one-third showed some concerns (Figure 2). At the level of individual studies, eight studies showed a low risk of bias and five of them showed some concerns. Domains with a high risk of bias included the index test, reference standard, and flow/timing (Figure 3).

Tissue sampling

**Figure 4:**
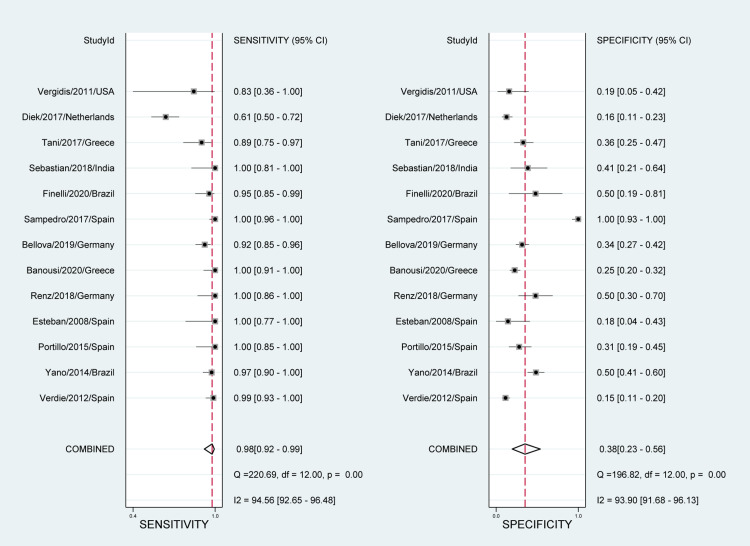
Paired forest plots of pooled sensitivity and specificity for tissue sampling Puig-Verdié/2013/Spain [30] Yano/2014/Brazil [31] Portillo/2015/Spain [32] Esteban/2008/Spain [33] Renz/2018/Germany [34] Banousi/2020/Greece [35] Bellova/2019/Germany [36] Fernández-Sampedro/2017/Spain [37] Finelli/2020/Brazil [38] Sebastian/2018/India [39] Tani/2017/Greece [40] Van Diek/2017/Netherlands [41] Vergidis/2011/USA [42]

The overall pooled estimates of sensitivity, specificity, PLR, NLR, and DOR of tissue sampling culture in diagnosing fracture-related infections were 98% (95% CI, 92% to 99%), 38% (95% CI, 23% to 56%), 1.6 (95% CI, 1.2% to 2.1%), 0.06 (95% CI, 0.01% to 0.30), and 25 (4 to 154), respectively (Table 2; Figure 4).

**Table 2:** Summary measures of diagnostic accuracy PLR: positive likelihood ratio; NLR: negative likelihood ratio; DOR: diagnostic odds ratio; AUC: area under the curve; CI: confidence interval

Parameter	Tissue sample culture			Sonication fluid sample culture		
	Estimate	95% LCI	95% UCI	Estimate	95% LCI	95% UCI
Sensitivity (%)	98	92	99	86	79	92
Specificity (%)	38	23	56	98	93	100
AUC (%)	84	81	87	96	94	98
PLR	1.6	1.2	2.1	49.1	11.5	210.9
NLR	0.06	0.01	0.30	0.14	0.09	0.22
DOR	25	4	154	353	78	1598

**Figure 5:**
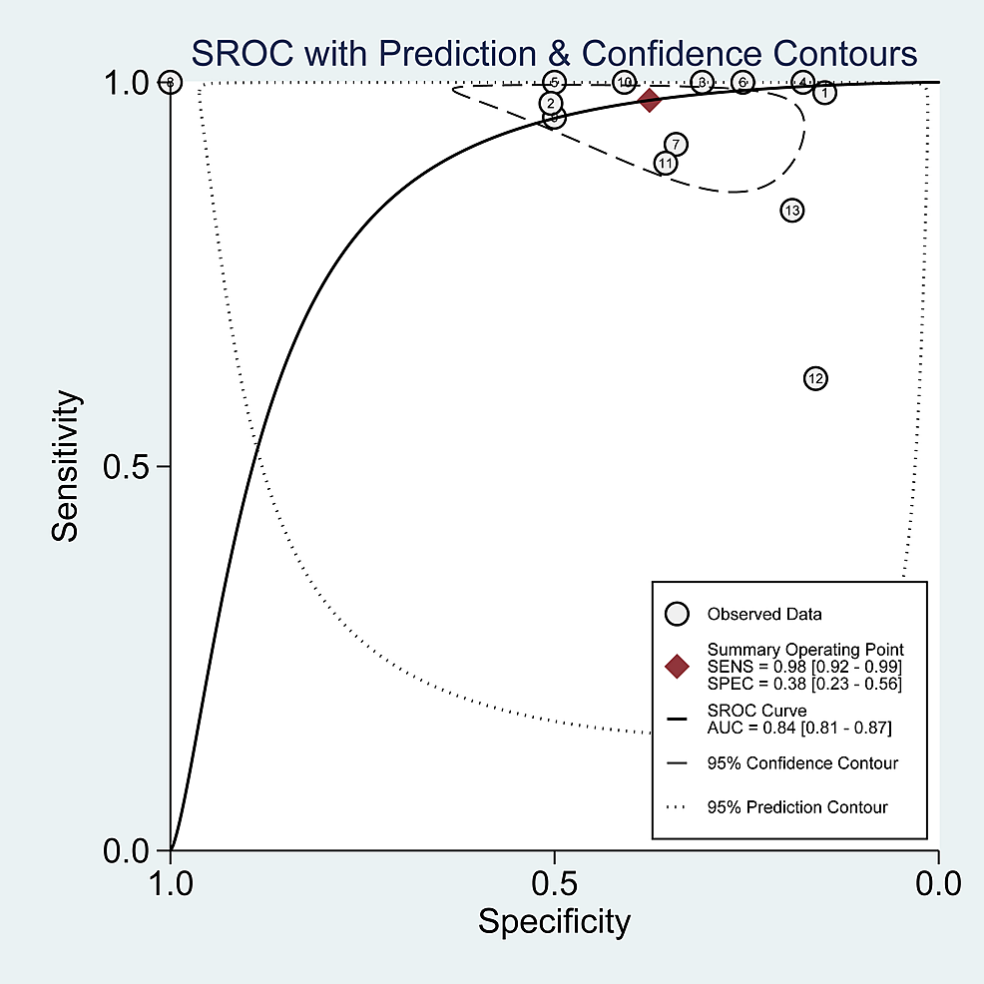
Summary receiver operating characteristic (SROC) plot for PET in tissue sampling. The number of studies that used PET is shown within each circle in tissue sampling PET: positron emission tomography

**Figure 6:**
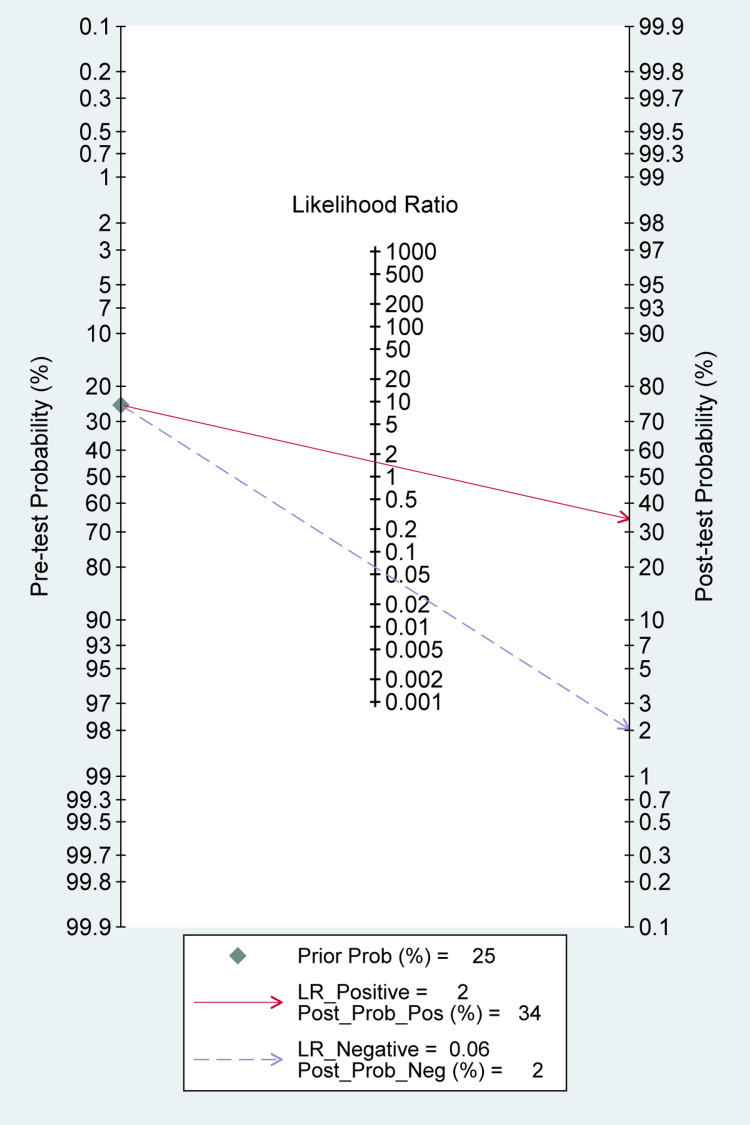
Fagan plot (Bayes Nomogram) for tissue sampling A vertical axis on the left with the prior log-odds, an axis in the middle representing the log-likelihood ratio, and a vertical axis on the right representing the posterior log-odds. Lines are then drawn from the prior probability on the left through the likelihood ratios in the center and extended to the posterior probabilities on the right.

The reported DOR denote that the OR for positive results among persons with fracture-related infections was approximately 25 times higher than the OR for positive results among persons with no infection. The SROC plot showed an area under the curve (AUC) of 84% (95% CI, 81 to 87%) (Figure 5). Fagan plot demonstrates that tissue sampling culture is informative, which raises the detection probability of a present fracture-related infection from 25% to 34% while lowering the probability of falsely detecting the disease to as low as 2% (Figure 6).

**Figure 7:**
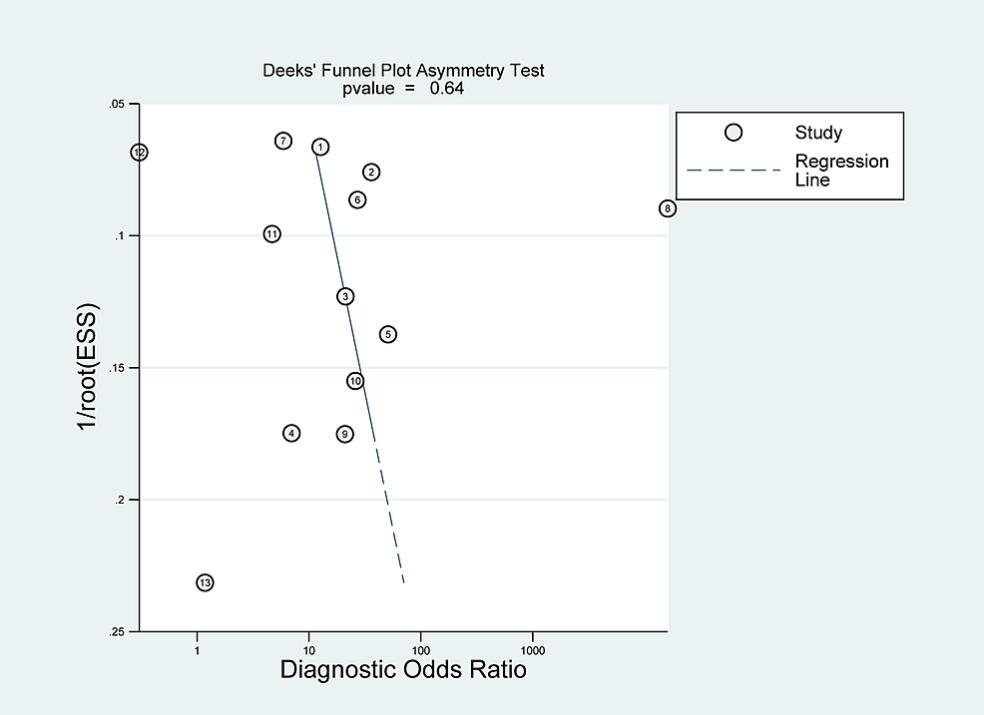
Funnel plot with superimposed regression line for tissue sampling analysis

**Figure 8:**
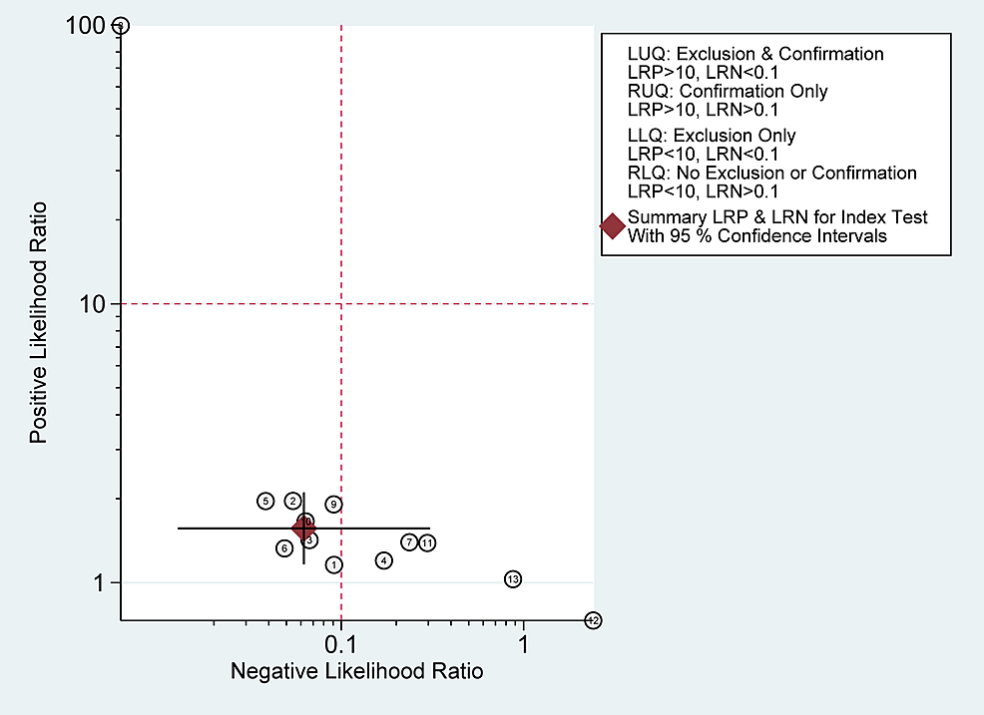
Likelihood ratio scattergram. It defines quadrants of informativeness based on established evidence-based thresholds for tissue sampling

**Figure 9:**
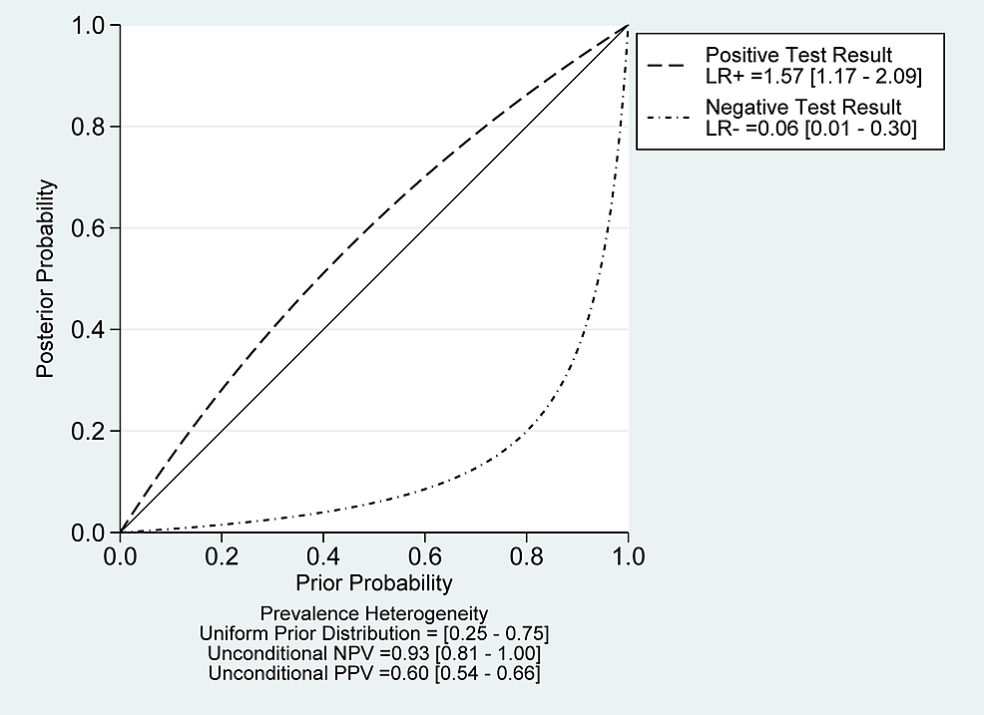
Probability modifying plot for tissue sampling analysis It shows the relationship between pre and post-test probability based on the likelihood of a positive (above diagonal line) or negative (below diagonal line) test result over the 0-1 range of pre-test probabilities.

Heterogeneity among the included studies was significant in sensitivity measures (I2 = 94.56%; P-value< 0.01) as well as specificity measures (I2 = 93.90%; P-value < 0.01) (Figure 4). However, no significant risk of bias was seen in Deek’s funnel plot asymmetry test (P-value= 0.64) (Figure 7). Sensitivity and specificity are summarized by the likelihood ratio scattergram (Figure 8) while Figure 9 reports the positive/negative predictive values by using the probability modifying plot to report the conditional probability of a disease.

Sonication fluid sampling

**Figure 10:**
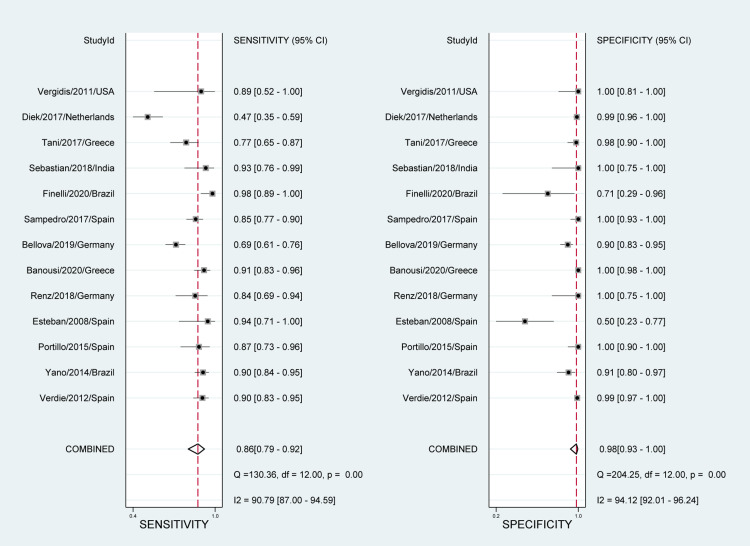
Paired forest plots of pooled sensitivity and specificity for sonication fluid sampling Puig-Verdié/2013/Spain [30] Yano/2014/Brazil [31] Portillo/2015/Spain [32] Esteban/2008/Spain [33] Renz/2018/Germany [34] Banousi/2020/Greece [35] Bellova/2019/Germany [36] Fernández-Sampedro/2017/Spain [37] Finelli/2020/Brazil [38] Sebastian/2018/India [39] Tani/2017/Greece [40] Van Diek/2017/Netherlands [41] Vergidis/2011/USA [42]

**Figure 11:**
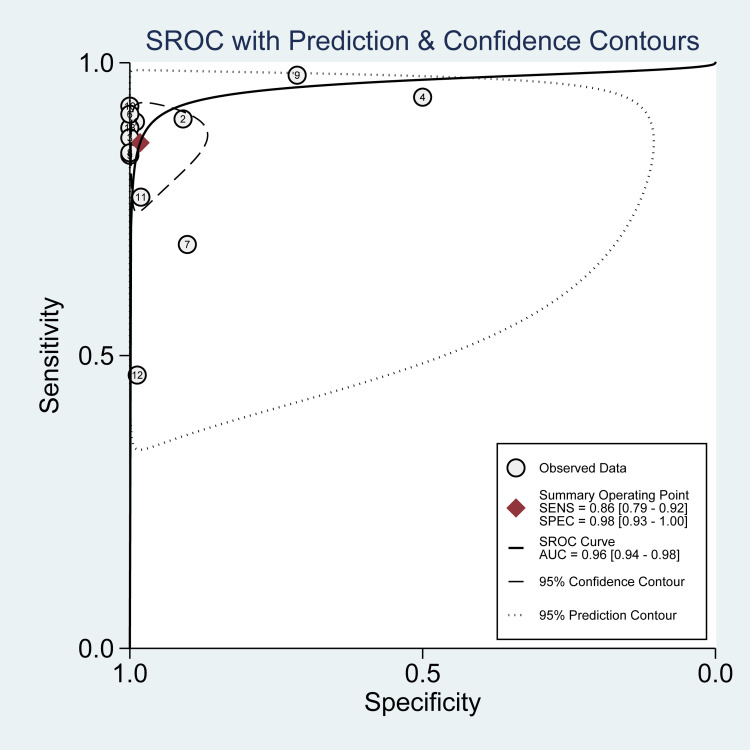
Summary receiver operating characteristic (SROC) plot for PET in sonication fluid sampling. The number of studies that used PET is shown within each circle in sonication fluid sampling PET: positron emission tomography

**Figure 12:**
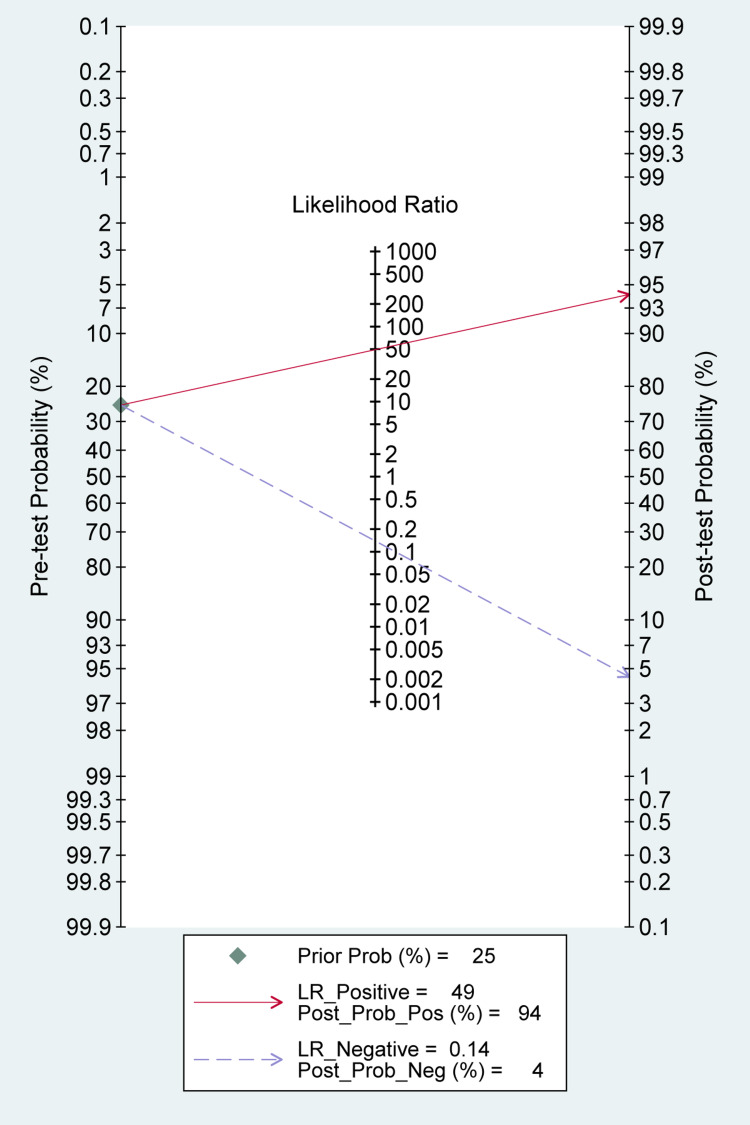
Fagan plot (Bayes nomogram) for sonication fluid sampling A vertical axis on the left with the prior log-odds, an axis in the middle representing the log-likelihood ratio, and a vertical axis on the right representing the posterior log-odds. Lines are then drawn from the prior probability on the left through the likelihood ratios in the center and extended to the posterior probabilities on the right

The overall pooled estimates of sensitivity, specificity, PLR, NLR, and DOR of sonication fluid sample culture in diagnosing fracture-related infections were 86% (95% CI, 79% to 92%), 98% (95% CI, 93% to 100%), 49.1 (95% CI, 11.5% to 210.9%), 0.14 (95% CI, 0.09 to 0.22), and 353 (78 to 1598), respectively (Table 2; Figure 10). The reported DOR denotes that the OR for positive results among persons with fracture-related infections was approximately 353 times higher than the OR for positive results among persons with no infection. The SROC plot showed an area under the curve (AUC) of 96% (95% CI, 94 to 98%) (Figure 11). Fagan plot demonstrates that sonication fluid sample culture is informative, raising the probability of detecting a present fracture-related infection from 25% to 94% and lowering the probability of falsely detecting the disease to as low as 4% (Figure 12).

**Figure 13:**
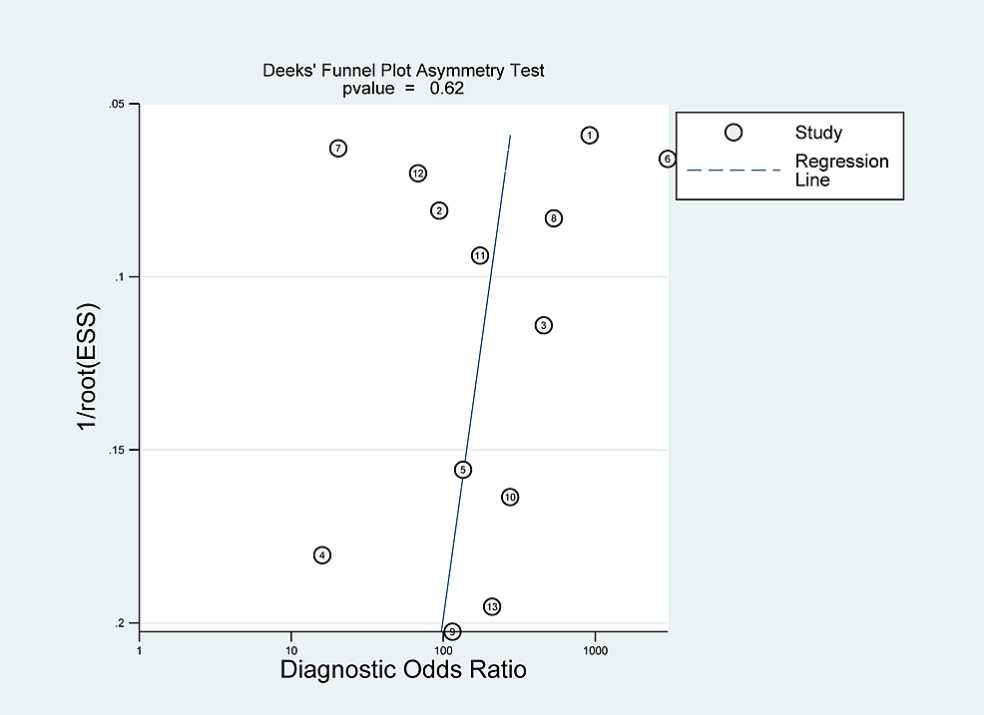
Funnel plot with superimposed regression line for sonication fluid sampling analysis

**Figure 14:**
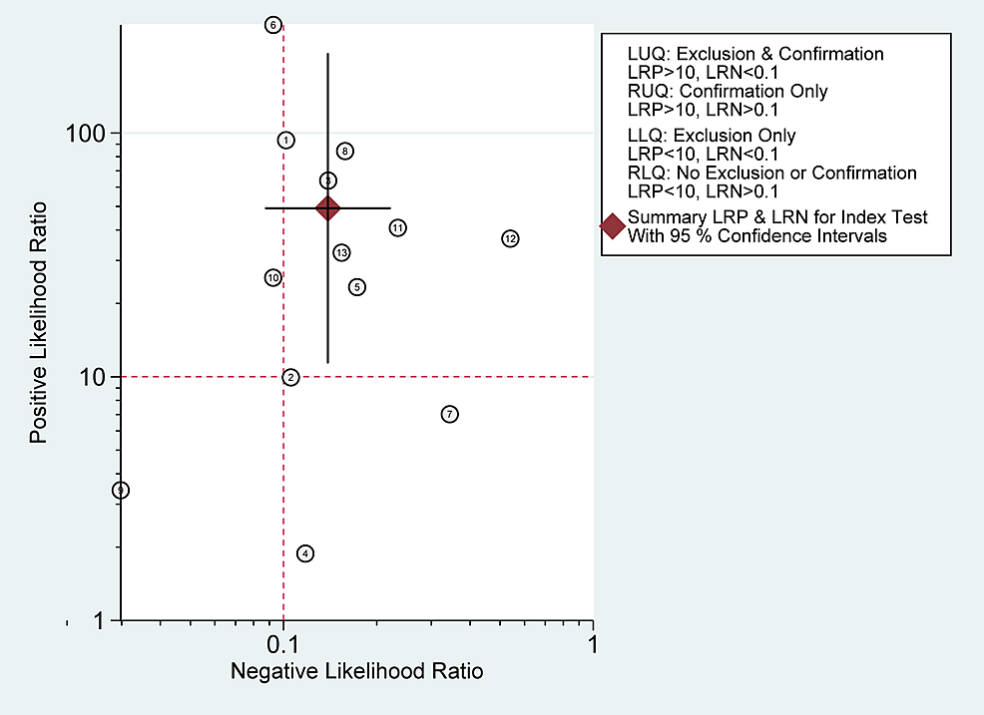
Likelihood ratio scattergram. It defines quadrants of informativeness based on established evidence-based thresholds for sonication fluid sampling

**Figure 15:**
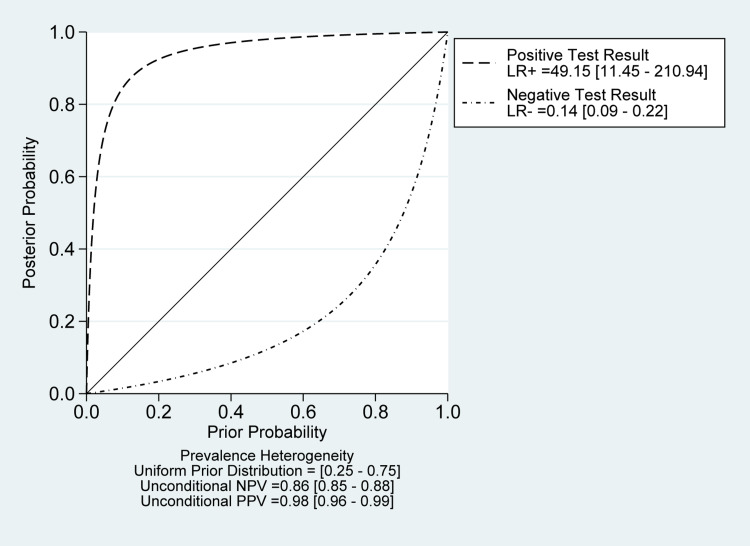
Probability modifying plot for sonication fluid sampling analysis It shows the relationship between pre and post-test probability based on the likelihood of a positive (above diagonal line) or negative (below diagonal line) test result over the 0-1 range of pre-test probabilities.

There was a significant heterogeneity among the included studies in sensitivity (I2 = 90.79%; P-value< 0.01) and specificity measures (I2 = 94.12%; P-value < 0.01) (Figure 10). However, the Deeks' funnel plot asymmetry test showed no significant risk of bias (P-value=0.62) (Figure 13). A likelihood ratio scattergram (Figure 14) showing the summary point of the likelihood ratios was obtained. The functions of mean sensitivity and specificity are demonstrated in the right upper quadrant, indicating that sonication fluid sample culture might be useful for only the confirmation of fracture-related infections (when positive). Figure 15 shows the given positive OR negative test and the so-called positive/negative predictive values in the probability modifying plot.

Discussion

Although tissue cultures are widely used as the standard tests for the diagnosis of FRI, no evidence in the literature could be found for validating this modality and whether other modalities can be superior to it [14]. This is the first meta-analysis that aims at validating tissue and sonication fluid sampling in the detection of FRI and suggesting which of these modalities is superior to the other, based on the results from 13 original investigations. We hope that our results would be used for drafting suitable protocols for the early, accurate diagnosis and proper management of FRI to avoid any possible complications.

The results from the analysis show that, although tissue culturing has a remarkably good sensitivity rate, the pooled specificity rate was 38%, and, therefore, the standardization of tissue culturing for the detection of FRI needs further reconsiderations. On the other hand, the estimated specificity rate for sonication fluid sampling was remarkably good, although the modality was relatively less sensitive than tissue culturing. Consequently, tissue culturing can be marked as a good negative test that should be preferred as a first-line diagnostic modality for the detection or exclusion of FRI while sonication fluid sampling can be marked as a good positive test that should be used for confirmation of the infection. We suggest that both modalities should be used, when applicable, to obtain the most diagnostic value available. Besides, based on our results, sonication fluid sampling can be considered superior to tissue culturing as the presented Fagan plot that was previously discussed.

Many differences and benefits could be compared to the previous systematic review by Onsea et al. [14]. At first, no meta-analysis was performed to validate the accuracy of both modalities based on data from the included studies, which was done in our study as previously discussed. Secondly, the authors of the review suggested that sonication fluid sampling is highly sensitive based on five investigations, however, our results indicate that it is less sensitive than tissue sample culture. Thirdly, the authors admitted that only three of the included studies compared both modalities in terms of FRI and although they showed that sonication fluid sampling is significantly superior to tissue sample culture regarding the accuracy of diagnosis, which is consistent with our findings, only one of these studies solely focused on FRI events. Meanwhile, significant heterogeneity was found despite not finding any risk of bias. We hereby suggest that future investigations with proper sample size and clear diagnostic protocols for FRI might be useful for further validation of our results. Previous evidence shows that sonication fluid sampling is useful and superior to tissue sample culturing in prosthetic joint infection events [36,43].

For increasing the sensitivity of sonication fluid sampling, previous approaches were suggested. A previous meta-analysis of 12 reports on the accuracy of sonication fluid sampling in prosthetic joint infection events suggested that conjugating this modality with centrifugation may increase the specificity, while the application of a 14-day anaerobic culturing system for the obtained samples might increase the sensitivity. Additionally, the authors showed that for increasing the specificity and sensitivity, 400-500 mL of Ringer’s solution should be applied [43]. Administration of antibiotics within 14 days before the sampling process can also increase the sensitivity of sonication fluid sampling, however, it may give false-negative results [31]. Additionally, organizing unified approaches to obtaining the samples might also enhance diagnostic accuracy and reduce the heterogeneity between studies [14]. Thus, sonication fluid sampling should be approached alongside tissue sample culturing to obtain the most accurate diagnosis.

Our study might have some limitations with regards to the estimated heterogeneity among the included studies and the limited population in some of them. Moreover, the absence of properly refined criteria might also have a significant effect on the estimated diagnostic accuracy. Consequently, we encourage that further approaches should be made for proper validation by using appropriate sample sizes with clear criteria.

## Conclusions

Sonication fluid sampling can be used to confirm FRI while tissue sampling can be used to exclude it. Tissue sampling is more sensitive while sonication fluid sampling is more specific and, therefore, the integration of both modalities is recommended to obtain the best results. Similar to the previous diagnosing prosthetic joint infections approaches, our results could help establish clear guidelines in diagnosing FRI. However, more studies are needed for further validation.
